# High frequency of vitamin D receptor gene polymorphism *Fok*I in Brazilian Type 1 diabetes mellitus patients with clinical autoimmune thyroid disease

**DOI:** 10.1186/s13098-016-0145-5

**Published:** 2016-03-22

**Authors:** Denise Barreto Mory, Monica Andrade Lima Gabbay, Eloá R. Rocco, Teresa Kasamatsu, Felipe Crispim, Walquíria Lopes Miranda, Sérgio Atala Dib

**Affiliations:** Endocrinology Division, São Paulo Federal University, Rua Botucatu, 740-Vila Clementino, São Paulo, SP CEP 04034-970 Brazil

**Keywords:** Type 1 diabetes, Autoimmune thyroid disease, Vitamin D receptor gene polymorphism

## Abstract

**Background:**

Polymorphisms of vitamin D receptor (VDR) gene have been studied as genetic markers of type 1 diabetes mellitus (T1DM) and some studies have reported associations with autoimmune thyroid disease. The aim of this study was to evaluate the relationship between VDR *Fok*I polymorphism (rs10735810), thyroid autoimmunity and thyroid dysfunction (TD) in Brazilian T1DM.

**Methods:**

One-hundred-eighty T1DM patients were evaluated for age, duration of diabetes (DDM), positivity to TPO Antibody (TPOA), GAD Antibody (GADA), IA2 Antibody (IA2A) and fasting serum C-peptide (FCP) according to diagnosis of TD. PCR–RFLP analyses were carried out for VDR polymorphism *Fok*I.

**Results:**

TPOA positivity (80.0 vs. 25.0 %, p < 0.001) and GADA positivity (56.0 vs. 30.3 %, p = 0.01) were higher in T1DM patients with TD with the same age and DDM than the group without TD, with no difference of FCP and IA2A positivity. We observed higher prevalence of VDR *Fok*I in T1DM with TD (ff and Ff 73.9 % with TD vs. 52.7 % without TD, p = 0.05). Positivity to TPOA and presence of *Fok*I polymorphism were significantly associated with the concurrence of TD in T1DM patients (OR 18.1; CI 3.7–87.0; p < 0.001).

**Conclusions:**

The VDR *Fok*I polymorphism (rs10735810) was associated to persistence of GADA, TPOA positivity and TD in Brazilian T1DM. Positivity to TPOA and VDR polymorphism *Fok*I were strongly associated with concurrence of T1D and TD. These data collaborate to understanding the joint susceptibility genes for TD in T1DM.

## Background

Type 1 diabetes mellitus (T1DM) often is associated to other autoimmune diseases, and the most frequent observed is autoimmune thyroid disease (AITD) [1–4].

Fifteen to 30 % of T1DM patients have autoimmune thyroid disease, as well as, 8 % of their first-degree relatives, showing an increased risk for autoimmune disease. Thyroid autoimmunity is increased in females and with longer duration of diabetes [4].

A possible explanation for the coexistence of thyroid and pancreatic autoimmunity can be found in share genetic background [5–9] and each disease is thought to be influenced by multiple susceptibility genes, as well as environmental factors [10].

Immune-modulating genes like HLA class II region gene, CTLA-4, CD40, PTNP22 and thyroid-specific genes [thyroglobulin and thyroid-stimulating hormone receptor (TSHR)] are involved in development of AITD [10, 11]. Of these, studies showed that HLA class II genes, CTLA-4, PTNP22 and FOXP3 contribute to susceptibility to both, T1DM and AITD [7, 8, 11–13]. All of them expressed on activated CD4+ and CD8+ with a role in T cell activation. This observation demonstrated that the majority of the genes associated with elevated disease risk relate to the function of the immune system [13, 14].

Vitamin D has been recognized for its effects on the immune system [15] and polymorphisms of vitamin D receptor gene (VDR) have been studied as genetic markers of T1DM [16] and of AITD [16–22].

In healthy populations, the *Bsm*I and *Taq*I bb genotype frequency was 2 % among Asians, 5 % among African Americans and 17 % among Caucasians while the *Apa*I AA genotype was 9, 44 and 28 % respectively. [23].

The VDR *Fok*I polymorphism in exon 2 leads to an alternative transcription initiation site resulting in a VDR protein with addition of three amino acids [24]. A functional role of this VDR polymorphism on the immune response has been previously described. It was observed in vitro that lymphocytes without VDR *Fok*I polymorphism proliferated more strongly, with more active immune response and, monocytes and dendritic cells without polymorphism produced higher levels of IL-12 p70 protein after stimulation [25].

Despite the well-known association among T1DM, thyroid auto antibodies and clinical AITD, there have been relatively few reports on the shared genetic susceptibility for these three events [5, 6, 13–22].

Our group had previously studied VDR gene polymorphism *Fok*I and T1DM, and we have not observed relationship with beta cell autoimmunity. Nevertheless, T1DM individuals with this polymorphism tended to have lower residual pancreatic beta cell function when they were compared with their pairs with the same duration of disease but without the polymorphism studied [26].

The aim of the present study was to evaluate the relationship between presence of VDR gene polymorphism *Fok*I (rs10735810), thyroid dysfunction (TD) and thyroid autoimmunity in a group of Brazilian T1DM.

## Methods

### Study population

We evaluated 180 patients with T1DM according to ADA criteria [27] attending the Diabetes Center of São Paulo Federal University, SP, Brazil. The study was approved by the Ethics Committee of São Paulo Federal University, Brazil (number 0814/03) and informed consent was obtained from the subjects’ parents.

### Clinical evaluation

Patients with T1DM from UNIFESP Diabetes Center were evaluated for age, sex, body mass index (BMI, kg/m^2^), age at diagnosis and time from diabetes mellitus diagnosis (TDD) and diagnosis of thyroid dysfunction. In those patients with TD we also evaluated age at diagnosis and time between development of diabetes and TD. The inclusion criteria used were: patients diagnosed with type 1 diabetes, according to ADA and with the presence of thyroid antibody or TSH and/or free T4 alteration.

Exclusion criteria were: patients who presented hypothyroidism due to other causes: iodine deficiency, post thyroid ablation.

Hypothyroidism and hyperthyroidism were clinically diagnosed and confirmed according to TSH levels. Thyroid dysfunction was diagnosed in the presence of a serum TSH alteration (normal 0.5–5.0 mUI/mL) with or without symptoms. Hypothyroidism was confirmed if TSH levels >5.0 mUI/mL and hyperthyroidism if TSH levels were bellow 0.5 mUI/mL. All patients with hypothyroidism were taking levothyroxine. Two patients with hyperthyroidism were treated with metimazol with remission of the disease after 1.5 and 1 year, and another one was still using 30 mg daily of this drug.

We considered presence of thyroid autoimmunity positivity for thyroid peroxidase antibodies (TPOA).

### Biochemical analysis

TSH was measured using an immunofluorometric assay developed in the Laboratory of Molecular Endocrinology of the Federal University of São Paulo, Brazil (normal range: 0.5–5.0 mUI/mL). TPOA were measured by an immunofluorometric assay (autoDelfia, Turku, Finland) and we considered the reference value <52 U/mL for the population studied. Glutamic acid decarboxylase 65 (GADA) and Insulinoma antigen-2 (IA2A) antibodies were measured by a radioimmunoassay (RSR Ltd, Cardiff, UK) and according to previous study of our group we considered the reference value <1.72 and 0.97 U/mL respectively [28]. Fasting serum C-peptide (FCP) was measured using an immunofluorometric assay (autoDelfia, Turku, Finland) with detection limit of 0.015 ng/mL. The intra-assay variation was 4.2 % (0.52–6.11 ng/mL) and inter-assay variation of 1.1 % (0.52 ng/mL), 3.4 % (6.11 ng/mL). Glycated hemoglobin (HbA1c) was measured in whole blood by HPLC (TOSOH, Japan; normal range 3.5–6.0 %).

### Genomic DNA extraction and genotyping

Genomic DNA was extracted from peripheral leukocytes using a commercial kit (PureGene Genome DNA Isolation Kit, Gentra Systems, Minneapolis, USA) and amplified by polymerase chain reaction (PCR). The forward primer for *Fok*I polymorphism (rs10735810, Genbank accession no AC004466) was 5′ AGCTGGCCCTGGCACTGACTCTGCTCT3′ and the reverse primer was 5′ ATGGAAACACCTTGCTTCTTCTCCCTC3′. The PCR conditions for the *Fok*I polymorphism were 94 °C for 5 min and 35 cycles using the following profile: 94 °C for 30 s, 60 °C for 30 s and 72 °C for 1 min and a final extension at 72 °C for 10 min. It was followed by restriction fragment length polymorphism according to previous reports for the VDR polymorphism *Fok*I [25]. Genotypes were determined by 1.8 % agarose gel electrophoresis and defined as lower case with the presence of restriction site and capital letters for its absence.

### Statistical analysis

Values are expressed as mean ± SD. Statistical analyses were performed with a SPSS for Windows version 13.0 (SPSS Inc. Chicago,Il, USA). We used Student’s t test, Mann–Whitney test (when variables not normally distributed) and χ^2^ tests to compare the demographics, clinical and laboratory characteristics of T1DM group according to presence or absence of TD. Logistic regression was performed in T1DM patients to assess the role of VDR *Fok*I polymorphism in the concurrence of T1DM and TD by including the genotypes of the VDR polymorphism. p values <0.05 were considered statistically significant. Hardy–Weinberg equilibrium was calculated to evaluate the gene and genotype frequencies expected and observed.

## Results

Screening for thyroid antibodies and function was performed in 180 T1DM with different time of clinical diabetes diagnosis.

TPOA was positive in 32.6 % (58/178) patients and TD was observed in 14 % (25/178). Twenty-two patients developed primary hypothyroidism and three hyperthyroidism after mean time of T1DM diagnosis of 3 years.

TPOA positivity (80.0 vs. 25.0 %, p < 0.001) were higher in T1DM patients with TD comparing to those patients that have not developed this dysfunction. Furthermore, the female gender (72.0 vs. 48.4 %, p = 0.02) and GADA positivity (56.0 vs. 30.3 %, p = 0.01) were also higher in T1DM patients with TD. There were no difference between groups related to age, age at diagnosis of diabetes, TDD, residual beta-cell function (FCP) and glycemic control (HbA1c).

Demographic, clinical, laboratory characteristics and prevalence of *Fok*I polymorphism are shown according to presence or absence of TD (Table 1).

**Table 1:** Demographic, clinical, laboratory characteristics and prevalence of VDR *Fok*I polymorphism of T1DM patients according to presence or absence of thyroid dysfunction (TD)

	With TD	Without TD	p
n (%)	25 (13.9)	155 (86.1)	
Gender (female %)	72.0	48.4	0.02
Age (years)	16.8 ± 7.1	17.1 ± 5.0	0.28
Age at diagnosis of diabetes (years)	10.0 ± 4.0	10.2 ± 5.1	0.80
TDDM (years)	6.9 ± 6.3	6.9 ± 5.2	0.72
Age at diagnosis of TD (years)	13.8 ± 6.5	–	
BMI (kg/m^2^)	21.3 ± 3.2	22.0 ± 3.6	0.18
TSH (mUI/mL)	19.1 ± 59.0	2.2 ± 1.4	0.00
TPO Ab positive (%)	80.0	25.0	<0.001
GAD Ab positive (%)	56.0	30.3	0.01
IA2 Ab positive (%)	36.0	43.1	0.50
FCP (ng/mL)	0.18 ± 0.29	0.19 ± 0.42	0.77
A1c (%)	9.3 ± 1.6	9.2 ± 1.8	0.75
ff/Ff genotypes (%)	73.9	52.7	0.05

Data are expressed as mean ± SD unless otherwise indicated

*TDDM* time of duration of diabetes, *FCP* fasting serum C-peptide

VDR polymorphism *Fok*I genotypes frequencies were in equilibrium of Hardy–Weinberg.

**Fig. 1:**
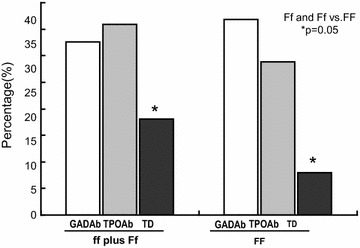
Frequency of positivity to GADA and to TPOA and prevalence of VDR *Fok*I polymorphism according to thyroid dysfunction

We observed higher frequency of homozygosis and heterozygosis for the VDR *Fok*I polymorphism in T1DM patients with TD comparing to those without (ff plus Ff genotypes 73.9 % with TD vs. 52.7 % without TD, p = 0.05; Fig. 1).

An analysis with interactive terms in a logistic regression with *Fok*I polymorphism and positivity to TPOA and to GADA was performed. T1DM patients with positivity to TPOA and *Fok*I polymorphism showed 18-fold increased risk of developing TD (OR 18.1, p < 0.001; Table 2), whereas for the concurrence of GADA titers and the presence of *Fok*I polymorphism the risk was of 8 times higher (OD 8.8, p = 0.009).

**Table 2:** ORs adjusted by logistic regression of associated variables for the association with thyroid dysfunction in T1DM

T1DM	B	p value	OR	95 % CI
Without TPOAb and without *Fok*I polymorphism			1.0	
Without TPOAb and with *Fok*I polymorphism	0.346	0.711	1.4	(0.2–8.8)
With TPOAb and without *Fok*I polymorphism	1.812	0.047	6.1	(1.0–36.6)
With TPOAb and with *Fok*I polymorphism	2.893	<0.001	18.1	(3.7–87.0)
Without GADAb and without *Fok*I polymorphism			1.0	
Without GADAb and with *Fok*I polymorphism	1.356	0.093	3.9	(0.8–18.9)
With GADAb and without *Fok*I polymorphism	1.433	0.114	4.2	(0.71–24.7)
With GADAb and with *Fok*I polymorphism	2.175	0.009	8.8	(1.71–45.2)

*TPOAb* thyroid peroxidase antibody

## Discussion

In a group of young T1DM patients and we found higher frequency of VDR *Fok*I polymorphism in individuals with thyroid auto antibodies without relation to age and duration of diabetes. The titers of thyroid autoantibodies and GADA were higher in T1DM with TD. Positivity to TPOA and presence of *Fok*I polymorphism were significantly associated with the concurrence of TD in T1DM patients. This is the first study that studied the association between those concomitant endocrine autoimmune diseases and VDR polymorphism in Brazilian T1DM patients.

T1DM and TD are autoimmune disease with common background. Both have the production of autoantibodies directed at the target organs (thyroid and pancreatic islets) and T cells-infiltration, resulting in their dysfunction and destruction. As autoimmunity progresses from initial activation to a chronic state, there is often an increase in the number of autoantigens targeted by T-cells and autoantibodies. T-cells activate additional autoreactive B-cells, and B-cells present additional epitopes from different proteins, until there is autoreactivity to diverse autoantigens [29]. Some authors consider the co-occurrence of T1DM and AITD in the same individual as one of the variants of autoimmune polyglandular syndrome type 3 (APS3s) [30].

Recent studies have evaluated shared genetic susceptibility to T1DM and Thyroid Autoimmune Disease [5–8, 12]. Nevertheless, studies with VDR polymorphisms did not evaluate individuals with these concomitant diseases [17–19]. Regarding these polymorphisms and thyroid disease distinct results have been reported. Japanese studies reported an association between VDR *Fok*I polymorphism, Hashimoto’s Thyroiditis and Graves’ disease [18, 19]. In German and Polish individuals this polymorphism was associated with Graves’ disease [19], but the same was not observed in patients from United Kingdom [20] and Tunisia [21]. In the Croatian population was described an association between VDR polymorphisms haplotypes and Hashimoto’s thyroiditis risk [22]. In T1DM, *Bsm*I polymorphism has been also linked to susceptibility to present the disease in Taiwanese, Japanese, Croatians and Southern Indians [31–33].

Our finding of higher frequency of VDR *Fok*I polymorphism in individuals with T1DM, thyroid auto antibodies and TD suggest that this polymorphism may be another loci that affect the development of both clinical diseases.

The susceptibility genes for T1DM and AITD identified to date, HLA class II, CTLA-4, PTNP22 and FOXP3 are involved in the immunological synapse and T cell activation [8, 34]. Van Etten et al. [25] had studied in vitro the role of *Fok*I polymorphism in autoimmunity and described that monocytes and dendritic cells without polymorphism produced higher levels of IL-12 p70 protein after stimulation. In BioBreeding rat, an animal model for AITD and diabetes, was observed an imbalance between cytokines secreted by Th1 and Th2 lymphocytes. IL-12 mRNA expression was increased in pancreatic islets and also in thyroid gland of those diabetic animals [35]. This is the major cytokine that induces a T helper 1 response, resulting in β cell destruction in T1DM [36]. Our study was not aim to evaluate the immune response but further studies are necessary to determine the relationship between cytokines, VDR polymorphism, thyroid autoimmunity and TD in T1DM patients.

In this study we observed a higher prevalence of VDR *Fok*I polymorphism in a group of T1DM Brazilian patients with a mean of 7 years of diabetes and 3 years of TD, which presented with persistence of GADA positivity and higher prevalence of TPOA positivity. Furthermore, positivity to TPOA and presence of VDR polymorphism *Fok*I were strongly associated with the presence of TD in T1DM patients. It was previously reported a tendency of higher frequency of thyroid auto antibodies in T1DM patients with HLA DR3/DR4 [37]. Also, a strong correlation between G allele of CTLA-4 gene polymorphism (rs 3087243) with TPOA was observed [38]. In Japan, two SNPs rs 2292399 in *ERBB3* and rs 2903692 in *CLEC16A* were associated with T1DM complicated with thyroid auto antibodies [6]. Concerning VDR polymorphisms, previous studies did not evaluate the relationship with thyroid auto antibodies [17–22].

An association between positivity to GADA and TPOA had been described in T1DM patients from different populations [3, 39–41]. Moreover, it was previously described an association between *PTNP22* 1858T genotype and GADA positivity in a group of T1DM patients with long disease duration, suggesting that this genetic variant may help define a subgroup of patients with persistence of GADA [42]. A single nucleotide polymorphism (rs 3087243) of CTLA4 was associated with AITD, but not with positivity of GADA [43]. In this study we observed that positivity to GADA and presence of *Fok*I polymorphism were significantly associated with the concurrence of TD in T1DM patients with an average of 7 years of disease.

Nevertheless, the VDR *Fok*I polymorphism might be another susceptibility loci for thyroid autoimmunity and an additional genetic marker that in association with auto antibodies might help identifying T1DM patients with higher risk of TD.

We observed hypothyroidism in 12.2 % of T1DM with a mean of 7 years of duration of diabetes, similar to observed in a prospective study that described a cumulative incidence of autoimmune thyroiditis of 14 % in pediatric T1DM patients at 10 years of diagnosis [44]. The diagnosis of T1DM preceded the development of TD in this group, as previously observed by our group [45]. In addition, 72 % of T1DM patients who developed TD were female and had also higher prevalence of thyroid autoimmunity, in accordance with previous studies [1, 2, 38, 46, 47].

In conclusion, the present study demonstrated that the VDR *Fok*I polymorphism (rs10735810) was associated to persistence and high titers of GADA, TPOA positivity and TD in Brazilian T1DM patients. The presence of VDR polymorphism *Fok*I was strongly associated with concurrence of T1DM and TD in this sample population. These data collaborate to understanding the joint susceptibility genes for TD in T1DM and could potentially improve the predictive value for this disease combination development.

## Abbreviations

VDR: vitamin D receptor
TD: thyroid dysfunction
DDM: duration of diabetes
T1DM: type 1 diabetes mellitus
AITD: autoimmune thyroid disease
TPOA: thyroid peroxidase antibody
GAD A: glutamic acid decarboxylase
IA2: islet antigen 2
FCP: fasting serum C-peptide
CTLA-4: cytotoxic T-lymphocyte –associated antigen
CD40: costimulatory protein
PTNP22: protein tyrosine phosphatase
TSHR: thyroid-stimulating hormone receptor
FOXP3: forkhead box P3
TSH: thyroid stimulating hormone
PCR: polymerase chain reaction
HLA: human leukocytes antigen
